# The interaction of PP1 with BRCA1 and analysis of their expression in breast tumors

**DOI:** 10.1186/1471-2407-7-85

**Published:** 2007-05-19

**Authors:** Sherry L Winter, Lucine Bosnoyan-Collins, Dushanthi Pinnaduwage, Irene L Andrulis

**Affiliations:** 1 Fred A. Litwin Centre for Cancer Genetics, Samuel Lunenfeld Research Institute, Mount Sinai Hospital, Toronto, Ontario, Canada; 2 Department of Pathology and Laboratory Medicine, Mount Sinai Hospital, Toronto, Ontario, Canada; 3 Division of Epidemiology and Biostatistics, Samuel Lunenfeld Research Institute, Mount Sinai Hospital, Toronto, Ontario, Canada; 4 Department of Molecular and Medical Genetics, University of Toronto, Toronto, Ontario, Canada; 5 Department of Laboratory Medicine and Pathobiology, University of Toronto, Toronto, Ontario, Canada

## Abstract

**Background:**

The breast cancer susceptibility gene, *BRCA1*, is implicated in multiple cellular processes including DNA repair, the transactivation of genes, and the ubiquitination of proteins; however its precise functions remain to be fully understood. Identification and characterization of BRCA1 protein interactions may help to further elucidate the function and regulation of BRCA1. Additionally, detection of changes in the expression levels of *BRCA1 *and its interacting proteins in primary human breast tumors may further illuminate their role in the development of breast cancer.

**Methods:**

We performed a yeast two-hybrid study to identify proteins that interact with exon11 of BRCA1 and identified Protein Phosphatase 1β (PP1β), an isoform of the serine threonine phosphatase, PP1. GST-pull down and co-immunoprecipitation assays were performed to further characterize this interaction. Additionally, Real-Time PCR was utilized to determine the expression of *BRCA1*, *PP1*α, β and γ in primary human breast tumors and normal breast tissue to identify alterations in the expression of these genes in breast cancer.

**Results:**

PP1 and BRCA1 co-immunoprecipitate and the region within BRCA1 as well as the specific PP1 interacting domain mediating this interaction were identified. Following mRNA expression analysis, we identified low levels of *BRCA1 *and variable levels of *PP1*α and β in primary sporadic human breast tumors. Furthermore, BRCA1, *PP1*β and PP1γ were significantly higher in normal tissue specimens (BRCA1 p = 0.01, *PP1*β: p = 0.03, *PP1*γ, p = 1.9 × 10^-6^) compared to sporadic breast tumor samples. Interestingly, we also identified that ER negative tumors are associated with low levels of *PP1*α expression.

**Conclusion:**

The identification and characterization of the interaction of BRCA1 with PP1 and detection of changes in the expression of *PP1 *and genes encoding other BRCA1 associated proteins identifies important genetic pathways that may be significant to breast tumorigenesis. Alterations in the expression of genes, particularly phosphatases that operate in association with BRCA1, could negatively affect the function of BRCA1 or BRCA1 associated proteins, contributing to the development of breast cancer.

## Background

The breast cancer susceptibility gene, *BRCA1*, is involved in the development of a significant proportion of familial breast and ovarian cancers and may also play a role in the development of sporadic breast cancer [1,2]. The BRCA1 protein has an amino terminal RING finger domain, potentially involved in ubiquitination, and two BRCA1 C-terminal domains (BRCT), which interact with a number of proteins involved in the transactivation of genes. The central portion of BRCA1, mainly encoded by exon11, contains two nuclear localization signals and interacts with proteins involved in DNA repair as well as the centrosomal protein, γ-tubulin [3]. Evidence suggests that BRCA1 functions as a scaffold, coordinating DNA repair [4], the transactivation of genes [5] and centrosome separation or maturation [6].

The phosphorylation of BRCA1 may be important for its function throughout the cell cycle. BRCA1 is hypophosphorylated during G1/S [7]; however, it becomes phosphorylated from S to G2/M phase. BRCA1 also interacts with kinases involved in cell cycle control and apoptosis such as the cyclin/cdk complexes [7], and a hypophosphorylated form of BRCA1 has been found at the centrosomes during mitosis [8].

The vertebrate serine/threonine protein phosphatase, PP1, has 3 isoforms: α, β (also known as δ) and γ that are highly conserved across their large catalytic domain, but are divergent at the amino- and carboxy-termini. Regulatory proteins bind to the unique carboxy termini of the PP1 isoforms to direct their isoform specific activities. For example, *PP1*γ knock-out mice have impaired spermiogenesis, indicating that the other PP1 isoforms are not entirely able to compensate for the loss of PP1γ; and the presence of polyploid spermatids in these mice suggests a defect in meiosis [9]. Furthermore, the PP1α isoform has also been found to associate with BRCA1 [10].

We have used a yeast two-hybrid assay to detect proteins that interact with exon11 of BRCA1. This large exon encodes roughly 60% of the protein, and we wished to identify potentially important interacting proteins outside of the intensely studied RING and C-terminal regions of BRCA1. The identification of PP1β as a BRCA1 interacting protein and characterization of the interaction of BRCA1 with PP1α, β and γ both *in vitro *and *in vivo*, provides new insights into the roles of both BRCA1 and the PP1 isoforms. These biochemical results led to analysis of the expression of *BRCA1 *as well as *PP1*α, β and γ, which has provided unique evidence for their potential deregulation in breast cancer. Disruption of the interaction of BRCA1 with PP1, or deregulation of its expression, could result in changes in the balance of kinase and phosphatase activities in the cell, leading to tumorigenesis.

## Methods

### Two-hybrid screening in yeast

Sequences from 900 to 4000 bp of BRCA1, were ligated into the DNA binding domain (DBD) yeast expression vector pAS1 (GAL4 _(1–147) _DNA-BD, TRP1, amp^r^). The yeast strains pJ69-4A (***MATa ****trp1-901, leu2-3, 112 ura3-52, his3-200, gal4*Δ, *gal80*Δ, *LYS2::gal1-HIS3, GAL2-ADE2, met2::GAL7-lacZ*) and Y187 (***MAT*α**, *ura3-52, his3-200, ade2-101, trp1-901, leu2-3, 112, gal4*Δ, *met*^-^, *gal80*Δ, *URA3::GAL1*_UAS_-*GAL1*_TATA_-*lacZ*) were utilized in this study. Yeast were initially transformed with the DBD-exon11 plasmid, followed by the Human Mammary Gland cDNA library cloned into the pACT2 Activation domain plasmid (Clontech). A total of 5 × 10^5 ^clones were tested.

### Co-immunoprecipitation

Coding sequences of PP1α, β or γ were ligated into the pCMVFlag (Sigma) expression vector. HEK293T cells were transfected with either of the PP1 vectors or the negative control pFLagLaf4 [11]. Cells were lysed using NETN buffer (150 mM NaCl, 1 mM EDTA, 20 mM Tris pH8.0, 0.5% NP40) along with the protease inhibitor cocktail 1 and phosphatase inhibitor cocktail 1 (Sigma); lysates were incubated with 1 μg/ml BRCA1 Ab1 and 3 (Oncogene) or 1 μg/mlM2-Flag (Sigma) rotating overnight at 4°C. 25 μl of protein G-agarose beads (Santa Cruz) were added and samples were incubated at 4°C for 2 hours. Antibodies used for Westerns were: 1 μg/ml BRCA1 Ab1 plus 1 μg/ml Ab3 (Oncogene) or 1 μg/ml M2 Flag (Sigma).

### Construction and expression of bacterial GST fusion proteins

Inserts of BRCA1 were generated using HiFiTaq Polymerase (Invitrogen), using the BRCA1 primers described in Scully *et al*. [12]. BR-4 V-A and F-A were generated using *in vitro *PCR mutagenesis [13] (Primers used were 1forward: GAAAGATCTGTAGAGAGTAGC, 1reverse (V-A): CAAAAGTGGCTTTTG GACTTTG, 1reverse (F-A): CATTCAGCAGTGACTTTTGGAC, 2forward (V-A): CCAAAAGCCACTTTTGAATGTG, 2forward (F-A): GTCACTGCTGAATGTGAAC, 2reverse: CCACTTCATTAGTACTGGAACC). pLysS bacterial cells, transformed with the BRCA1 constructs and induced for protein expression, were lysed using B-Per bacterial cell lysis reagent (Pierce) plus 1 mM PMSF and DNAse (200U/ml, Invitrogen) according to manufacturer's directions. Lysates were incubated with 50 μl of a 50% GST slurry overnight at 4°C. The amount of GST-bound protein was determined by comparison with known amounts of BSA loaded onto the gel, with amounts ranging from 0.1ug to 2ug of BSA.

### *In vitro *GST-interaction studies

1 μg of GST-bound protein was added to HEK293T cell lysate (1 mg of protein per reaction), and incubated rotating at 4°C for 2 hours. GST pellets were washed 3X with 1 mL of NETN + protease and phosphatase inhibitors (Sigma) and GST-bound proteins were eluted using SDS PAGE loading dye. Samples were electrophoresed on a 12% SDS-PAGE gel and transferred to a nitrocellulose membrane. Immunoblots were probed with 1 μg/ml of the PP1 E-9 antibody (Santa Cruz) and bands were visualized using a Fluor-S MultiImager (BioRad). Binding of PP1 to BR-4 V-A or F-A was compared with binding of PP1 to wild-type BR4 in order to determine the relative degree of interaction.

### Tissue samples

Twenty-nine tumors from sporadic breast cancers were obtained as part of a prospective study of molecular alterations in auxiliary node-negative disease [14]. Following frozen section diagnosis of invasive cancer, tumor specimens were sampled by a pathologist and immediately snap frozen and stored in liquid nitrogen. DNA and RNA have previously been extracted from these tumors by conventional techniques [15]. Normal breast mRNA was obtained, by a pathologist, from adjacent tissue surrounding breast tumors and was isolated as outlined above.

### Real-time PCR expression analysis

cDNA was reverse transcribed from 250 ng of RNA using MMLV-RT and random hexamers as directed (Invitrogen). cDNA was amplified using the Applied Biosystems Taqman Universal PCR Master Mix (no UNG) (cat# 4324018), 8 μl of diluted cDNA (1 μl of cDNA + 7 μl of ddH_2_0), 1 μl of 20 × Assay on Demand gene expression assay mix (HPRT1 control primer/probe mix) and 1 μl of 20 × Assay on Demand gene expression assay mix (test primer/probe mix- catalogue numbers are listed below). Thermal cycling consisted of a hold at 95°C for 10 minutes followed by 40 cycles of 95°C for 15 seconds and 60°C for 60 seconds, using the ABI PRISM 7900HT Sequence Detection System (Applied Biosystems (ABI)). Standard curves of serial dilutions ranging from 3 × to 0.005 × of a pool of cDNA were used for quantification, and slopes generated by the standard curves ranged from 3.3 to 3.6. Test probes were conjugated to the FAM™ fluor (Applied Biosystems), and the HPRT1 endogenous control probe was conjugated to the VIC™ fluor (Applied Biosystems). The HPRT-1 internal control, which has been determined by our lab and others to have little variation between breast tumors [16,17], was chosen to normalize for variations in mRNA amount and quality between tumors. We compared the expression levels of the genes to that of the HPRT-1 housekeeping gene for each sample, and all mRNA/HPRT-1 expression levels were approximately one when comparing a pool of cell line cDNA for each experiment to prevent variation between experiments. For our purposes, mRNA/HPRT-1 ratios of less than 0.5 × the mean level of expression were considered to be under-expressed, while mRNA/HPRT-1 ratios of greater than 2 × the mean level of expression were considered to be over-expressed. The test and control probes were multiplexed, and analysis was performed on ratios of the test quantity mean values to control quantity mean values for each sample. ABI SDS2.1 software was used to analyze the results. The primer/probe pairs used for the experiments were purchased from ABI (cat#: *PP1*α: Hs00267568-m1, *PP1*β: Hs00160343-m1, *PP1*γ: Hs00160351-m1, *BRCA1*: HS 00173233-m1, *HPRT-1*: 4326321E_(endogenous control)).

### Statistical analysis of tumor characteristics and their association with PP1α and PP1β expression

A descriptive analysis was performed, comparing frequency distributions of tumor characteristics between *PP1 *groups ('high': greater than 2.2 or 'low': less than or equal to 2.2), using contingency tables. The median level of expression of PP1α, in all tumors analyzed (familial (not shown) and sporadic), was used to establish the level for high vs. low *PP1 *expression. Association of each characteristic with *PP1 *expression was investigated by Fisher's exact test [18].

All statistical analyses were performed using SAS statistical software, version 8.2 (SAS Inc., Cary, NC, USA). p < 0.05 was considered to be statistically significant.

### Cell culture conditions and transfections

Transfections were performed using Fugene transfection reagent (Roche) according to manufacturer's directions. HEK293T cells were acquired from the American Type Tissue Collection and were grown under their recommended conditions.

## Results

### Association of BRCA1 with PP1 *in vitro*

We performed a yeast two-hybrid assay to identify proteins that interact with exon11 of BRCA1. In this study, 9 putative positives were identified including PP1β, an isoform of the serine/threonine phosphatase PP1 (not shown). These initial yeast two-hybrid studies led to further examination on the interaction of BRCA1 with PP1β.

**Figure 1:**
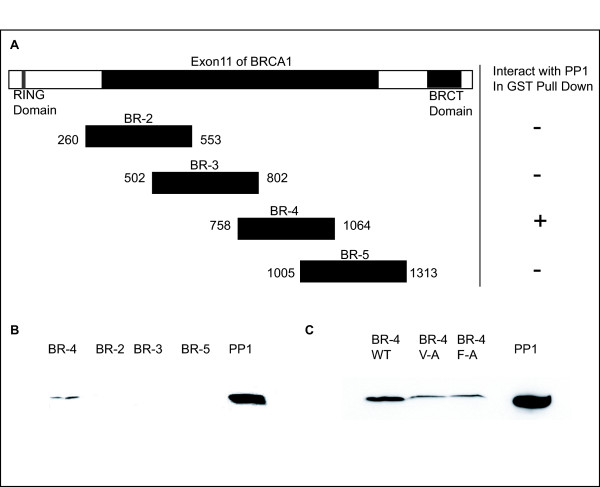
**GST-pull down assay to identify the region of BRCA1 interacting with PP1**. **(A) **Fragments used in GST pull down assays (BR 2 to 5) are diagrammed. **(B) **Gel depicting co-precipitation of GST-bound BR-4 with PP1. Following incubation of GST-BRCA1 proteins with equal amounts of cell lysate, a western blot was performed and probed with an antibody to the catalytic region of PP1. **(C) **Analysis of the effect of mutations of the KVTF PP1 interacting domain on the BRCA1- PP1 interaction. GST-bound-BR4 V-A and GST-bound-BR4 F-A binds PP1 with decreased intensity, compared to WT GST-bound-BR4.

Overlapping fragments of BRCA1 (Figure 1A) were created to identify the region of BRCA1 required to interact with PP1 and we determined that BR-4 (amino acids 758 to 1064) mediated the interaction with PP1 (Figure 1B). Sequence analysis of the BR-4 fragment identified a putative PP1 interacting domain, (K/R)/(V/I)/XF [19,20], which is present in several regulatory proteins that interact with PP1. Similarly to other PP1 interacting domains [19,21], there are 2 positively charged amino acids upstream (^893^**KK**^894^), and a group of negatively charged amino acids downstream (^902^**E**C**E**QK**EE**^908^) of the BRCA1 KVTF sequence, further supporting its classification as a putative PP1 interacting domain.

Mutation of the valine to alanine, or phenylalanine to alanine, within the (K/R)/(V/I)/XF PP1 binding domain, has been shown to affect the binding of PP1 with other interacting proteins [22]. When BR-4 containing a V-A or F-A alteration was analyzed for its ability to interact with PP1, a significant decrease was observed in the amount of PP1 that interacted with the BR-4 V-A or F-A GST fusion protein compared to the wild type BR-4 fragment (Figure 1C), indicating that this sequence is required to mediate the interaction between BRCA1 and PP1.

### Association of BRCA1 and PP1 *in vivo*

To establish that the association between BRCA1 and PP1 is a physiological interaction, HEK293T cells were transfected with Flag-epitope tagged PP1 α, β or γ or pFLAG Laf4 [11]. Laf4 was chosen as a negative control since it is expressed in the nucleus, but was not anticipated to interact with BRCA1. We hypothesized that, although BRCA1 was shown to interact with PP1β in the two-hybrid screen, it might be capable of interacting with all three PP1 isoforms PP1 due to their high degree of sequence similarity.

**Figure 2:**
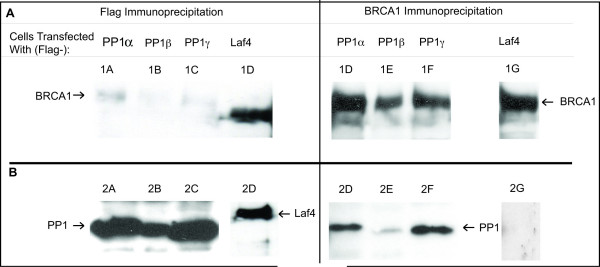
**Coimmunoprecipitation of BRCA1 and PP1**. HEK293T kidney cells were transfected with vectors encoding untagged BRCA1 under the control of a CMV promoter, and vectors encoding Flag-PP1α, β, γ or Flag-Laf4. **(A) **A western blot probed with BRCA1 shows that immunoprecipitation of protein with an antibody against the Flag-PP1α, β or γ proteins, but not Laf4, co-immunoprecipitates BRCA1 (Lanes 1A-1D). It should be noted that the band observed slightly lower than BRCA1 in lane 1D is a non-specific background band. Lanes 1E-1H show immunoprecipitation of BRCA1 using antibodies against the amino and carboxy termini of BRCA1. **(B) **A western blot probed with an antibody against the Flag epitope. Lanes 2A-2D indicate immunoprecipitation of the Flag-epitope tagged PP1α, β or γ or Flag-Laf4. Lanes 2E-2G show co-immunoprecipitation of Flag-PP1α, β or γ with antibodies against BRCA1, and lane 2H shows a lack of coimmunoprecipitation of the negative control Flag-Laf4 by BRCA1.

Protein was immunoprecipitated using antibodies against BRCA1, or an antibody against the Flag epitope to immunoprecipitate Flag epitope tagged PP1α, β, or γ. BRCA1 coimmunoprecipitated all three PP1 isoforms, and conversely, PP1 α, β and γ coimmunoprecipitated BRCA1 (Figure 2), indicating that the interaction between BRCA1 and PP1 is specific.

### Analysis of the expression of *PP1*α, β and γ, and genes encoding other BRCA1 associated proteins in breast tumors

Our investigations into the interaction of BRCA1 with PP1 led us to examine the expression levels of BRCA1, *PP1*α, β and γ in primary human breast tumors. We determined that the expression levels of *PP1*α and β were variable in breast tumors, with several tumors exhibiting very low or high expression, relative to the mean level of expression observed for these genes (Table 1). In contrast, *PP1*γ levels were less variable, with most tumors exhibiting expression levels that were not notably outside the mean level of *PP1*γ expression. Interestingly, when analyzing levels of *PP1*α, β and γ in normal breast tissue (Table 2), we observed that levels of *PP1*β and γ were significantly higher in normal tissue specimens (*PP1*β: p = 0.03, *PP1*γ, p = 1.9 × 10^-6^) compared to sporadic breast tumor samples. A similar trend for decreased *PP1*α expression was also observed, although significance was not reached for this isoform.

**Table 1:** Distribution of gene expression in Primary Sporadic Human Breast Tumors

**Gene Name**	**Number of Tumors (N)**	**Mean expression level (M)**	**N < 0.5 × M**	**N > 2 × M**
**PP1α **	24	3.1	4 (17%)	2 (8.4%)
**PP1β **	26	2.8	5 (19 %)	5 (19 %)
**PP1γ **	23	1.3	1 (4.3%)	1 (4.3%)
**BRCA1**	25	0.6	7 (28%)	2 (8%)

^1^test gene/HPRT-1 expression ratios as measured by quantitative real-time PCR

**Table 2:** Gene Expression Levels in Normal Breast Tissue Compared to Sporadic Breast Tumors

**Name of Gene**	**Tissue Type**	**Mean Expression Level**^1^	**Significance (p)**
**BRCA1**	Normal	0.9	0.01
	Sporadic	0.6	
**PP1α **	Normal	3.6	0.29
	Sporadic	3.1	
**PP1β **	Normal	3.9	0.03
	Sporadic	2.8	
**PP1γ **	Normal	3.2	1.9 × 10^-6^
	Sporadic	1.3	

^1. ^mRNA/HPRT-1 expression ratios as measured by quantitative real-time PCR, relative to the pool of cDNA

*BRCA1 *has previously been reported to have a decreased level of expression in tumors, relative to normal breast tissue [2]. We also observed that 28% of the tumors we analyzed had low levels of *BRCA1*, and 8% of those tumors had extremely low to non-detectable levels of *BRCA1 *expression. Not surprisingly, *BRCA1 *expression levels in normal breast tissue were significantly higher those seen in sporadic breast tumors (p = 0.01).

### Tumor characteristics and association with *PP1*α expression

To identify an association with the characteristics of the breast cancer cases used in this study and their level of *PP1*α and β expression, contingency-table Fisher's exact tests were performed. The group of breast tumors with a 'low' level of *PP1*α mRNA was compared to those with 'high' *PP1*α expression. Although no correlations were observed in age, menopausal status, tumor size, histologic grade, PgR status and lymphatic invasion, we did detect an association between low *PP1*α expression and ER status (*p *= 0.02). Tumors with 'low' *PP1*α levels were more likely to be negative for ER receptor than those with 'high' *PP1*α levels (87% vs. 25%). No correlations were observed between 'high' or 'low' *PP1*β expression and any of the tumor characteristics outlined above.

## Discussion

BRCA1 interacts with a large number of proteins, and may function as a scaffold protein to bring cell cycle or DNA repair processes together, but the mechanisms by which BRCA1 functions in these processes have yet to be fully elucidated. We performed a yeast two-hybrid study to identify proteins that interact with exon11 of BRCA1. The region of BRCA1 encoded by exon 11 is known to interact with a number of proteins involved in DNA repair [23], as well as γ-tubulin [3] and several kinases including Aurora-A kinase [24] and ChkII [25]. Identification of additional interacting partners, particularly ones that could modify the activity of a BRCA1 through changes in phosphorylation, may further aid in clarifying its function and regulation. In this yeast two-hybrid study, we identified the serine/threonine phosphatase PP1β as a BRCA1 interacting protein, which could have important consequences on both the activity of BRCA1 and the regulation of PP1β activity.

PP1 has 3 isoforms encoded by different genes that are 97% conserved across their catalytic domains and distinct roles for each isoform have yet to be determined. When we coimmunoprecipitated Flag-epitope tagged PP1α, β or γ with BRCA1, we observed that all 3 isoforms interacted with BRCA1. Additionally, we have identified the functional PP1 interacting domain within BRCA1. This domain is found in other PP1 regulatory proteins, suggesting that BRCA1 may regulate the activity of PP1 and could act as a scaffold protein to promote the dephosphorylation of BRCA1 associated proteins by PP1.

The expression levels of *BRCA1 *and the *PP1 *isoforms were analyzed in primary human breast tumors. Low levels of *BRCA1 *mRNA were identified, consistent with decreased expression rather than mutation as a method for *BRCA1 *inactivation in these tumors. Additionally, we observed variable levels of expression for *PP1*α and β, but not *PP1*γ. Interestingly, decreased levels of *PP1*β and γ were identified when comparing their gene expression levels from normal breast tissue with expression levels from sporadic breast tumors (Table 2). This decreased expression may lead to perturbations of PP1 protein levels, altering the balance of kinase and phosphatase activities acting on specific substrates and potentially disrupting important cellular functions.

The use of primary human breast tumors allowed us to examine correlations between tumor and patient characteristics with *PP1*α or β expression. Interestingly, we observed a statistically significant association between the level of *PP1*α expression and estrogen receptor (ER) status using our small sample of 24 sporadic tumors, which will need to be investigated further in a larger sample set. ER turnover is affected by a basal level of phosphorylation that is maintained through a balance of kinase and phosphatase activities [26]. Activation of protein phosphatase PP2A has recently been shown to increase the level of ER mRNA stability [27]. Our results indicate that tumors with low expression of *PP1*α are more likely to be ER negative than tumors with high expression of *PP1*α (87% vs. 25%) and it is possible that PP1α has a role in ER mRNA stability, similarly to that of PP2A.

## Conclusion

We have characterized the interaction of PP1 with BRCA1 and have identified a PP1 binding domain within BRCA1 that is necessary for this interaction to occur. Furthermore, expression of the *PP1 *isoforms as well as several genes encoding BRCA1 interacting proteins was analyzed in primary human invasive breast tumors. We detected low levels of *BRCA1 *expression in 28% of tumors, respectively, and variable levels of *PP1*α and β. Moreover, significant decreases in expression were observed for *BRCA1*, *PP1*β and PP1γ when comparing normal breast tissue with invasive breast cancers. Additionally, a significant association of *PP1*α levels with ER status in breast tumors was identified that could lead to additional studies into the effect of PP1α on ER mRNA stability and its role in the development of breast cancer. The studies presented here provide evidence for an important role for PP1 in the development of breast cancer, possibly through its association with BRCA1, and suggest that deregulation of the balance of kinase and phosphatase activity in the cell may be an important component of breast tumorigenesis.

## Abbreviations

BRCA1: Breast cancer susceptibility gene 1, BRCT (BRCA1 Carboxy-terminal domain), AD: Activating Domain, DBD: DNA binding domain, MEF: Mouse embryonic fibroblast, PP1: Protein Phosphatase 1

## Competing interests

The author(s) declare that they have no competing interests.

## Authors' contributions

SLW performed the biochemical analysis for the interaction of BRCA1 and PP1 and designed the expression studies, interpreted results and drafted the manuscript. LB-C performed the Real-Time expression analysis of gene expression, DP performed statistical analysis on the results and ILW supervised all work and aided in the drafting of the manuscript. All authors have read and approved the final manuscript.

## Pre-publication history

The pre-publication history for this paper can be accessed here:


